# In Silico Prediction of Intestinal Permeability by Hierarchical Support Vector Regression

**DOI:** 10.3390/ijms21103582

**Published:** 2020-05-19

**Authors:** Ming-Han Lee, Giang Huong Ta, Ching-Feng Weng, Max K. Leong

**Affiliations:** 1 Department of Chemistry, National Dong Hwa University, Shoufeng, Hualien 974301, Taiwan; 610512018@gms.ndhu.edu.tw (M.-H.L.); 810812203@gms.ndhu.edu.tw (G.H.T.); 2 Department of Basic Medical Science, Center for Transitional Medicine, Xiamen Medical College, Xiamen 361023, China; cfweng-cfweng@hotmail.com

**Keywords:** intestinal permeability, passive diffusion, active transport, in silico, quantitative structure–activity relationship, hierarchical support vector regression

## Abstract

The vast majority of marketed drugs are orally administrated. As such, drug absorption is one of the important drug metabolism and pharmacokinetics parameters that should be assessed in the process of drug discovery and development. A nonlinear quantitative structure–activity relationship (QSAR) model was constructed in this investigation using the novel machine learning-based hierarchical support vector regression (HSVR) scheme to render the extremely complicated relationships between descriptors and intestinal permeability that can take place through various passive diffusion and carrier-mediated active transport routes. The predictions by HSVR were found to be in good agreement with the observed values for the molecules in the training set (*n* = 53, *r*^2^ = 0.93, $q_{\mathrm{CV}}^{2}$ = 0.84, RMSE = 0.17, *s* = 0.08), test set (*n* = 13, *q*^2^ = 0.75–0.89, RMSE = 0.26, *s* = 0.14), and even outlier set (*n* = 8, *q*^2^ = 0.78–0.92, RMSE = 0.19, *s* = 0.09). The built HSVR model consistently met the most stringent criteria when subjected to various statistical assessments. A mock test also assured the predictivity of HSVR. Consequently, this HSVR model can be adopted to facilitate drug discovery and development.

## 1. Introduction

Oral administration is the predominant route for medication that can be manifested by the fact that ca. 56% of unique drugs approved by FDA in 2018 were orally administrated [1]. Accordingly, drug absorption is one of critical absorption, distribution, metabolism and excretion, and toxicity (ADME/Tox) factors that should be taken into consideration in the process of drug discovery and development as well as practical applications [2]. For instance, curcumin, which is the major constituent of the spice turmeric (*Curcuma longa*), has a great beneficial potential in treating cancer, diabetes, osteoarthritis, antianxiety, and even novel coronavirus disease 2019 (COVID-19) [3,4] and yet its practical clinical applications are very limited mainly due to its poor absorption [5]. Clinically, tuberculosis (TB) is one of the leading causes of death globally, especially for HIV/AIDS patients [6], and the survival of extremely ill TB patients is diminished due to the poor absorption of anti-TB agents [7].

Drug absorption mainly relies on solubility and intestinal permeability [8], which is also termed as intestinal absorption [9], since oral drugs must permeate the gastrointestinal barrier before they can be absorbed by the bodies [9]. In fact, solubility and permeability have been adopted by the biopharmaceutics drug disposition classification system (BDDCS), which suggests that the intestinal permeability rate is closely correlated with the extent of metabolism [10] Nevertheless, intestinal permeability is an extremely complicated process since drugs can pass through the intestinal epithelium to enter blood vessel by active transport as well as passive diffusion, as illustrated by Figure 3 of Dahlgren and Lennernäs [11]. Mechanistically, the active transport can be mediated by two superfamilies expressed in the intestine, namely the influx transporters of the solute carrier (SLC) family and the efflux transporters of the ATP-binding cassette (ABC) family, whereas the passive diffusion can take place through the transcellular and/or paracellular routes [12].

In addition, the ABC transporters including P-glycoprotein (P-gp, MDR1, *ABCB1*), breast cancer resistance protein (BCRP, *ABCG2*), MRP2 (*ABCC2*), MRP3 (*ABCC3*), MRP4 (*ABCC4*), MRP5 (*ABCC5*), MRP6 (*ABCC6*), MRP7 (*ABCC10*), MRP8 (*ABCC11*), and MRP9 (*ABCC12*) [13], and the SLC transporters involving peptide transporter 1 (PepT1, *SLC15A1*), concentrative nucleoside transporter 1 (CNT1, *SLC28A1*), concentrative nucleoside transporter 2 (CNT2, *SLC28A2*), equilibrative nucleoside transporter (ENT2, *SLC29A2*), organic cation transporters 1 (OCT1, *SLC22A1*), organic cation/carnitine transporter 1 (OCTN1, *SLC22A4*), organic cation/carnitine transporter 2 (OCTN2, *SLC22A5*), monocarboxylate Transporter 1 (MCT1, *SLC16A1*), organic anion transporting polypeptide 2B1 (OATP2B1, *SLC02B1*), serotonin transporter (SERT, *SLC6A4*), and apical sodium-dependent bile acid transporter; (ASBT. *SLC10A2*) [14] can be found in the intestine. Their expression levels can be different in varied segments of intestine [15,16].

Of various in vitro assay systems to measure intestinal permeability, human colorectal adenocarcinoma cells (Caco-2), Madin−Darby canine kidney cells (MDCK), and parallel artificial membrane permeability assay (PAMPA) are commonly used [9], and they can be affected by factors such as cell line types and cultured conditions. The in situ single-pass intestinal perfusion (SPIP) model is the most prevalent assay [17] that normally measures effective permeability (*P*_eff_) of the gastrointestinal (GI) tract segments, namely duodenum, jejunum, ileum, and colon, in human, rat, and mouse [18]. The parameter *P*_eff_, which is expressed as cm/s, can be calculated by

(1) $$P_{\mathrm{eff}}=\frac{-Q\ln\left(C_{\mathrm{out}}^{\prime }/C_{\mathrm{in}}^{\prime }\right)}{A}$$

where *Q* is the perfusion buffer flow rate; $C_{\mathrm{out}}^{\prime }$ and $C_{\mathrm{in}}^{\prime }$ are the outlet and inlet solute concentrations, respectively; and *A* represents the surface area within the intestinal segment that can be computed by the radius of the intestinal segment (*R*) and the length of the perfusion intestinal segment (*L*) [19],

(2) A=2πRL

When compared with in vitro assays, in vivo tests provide a closer to real-life environment, but they are costly, time consuming, and sometimes inhumane, and are subjected to discrepancies by a number of factors such as individual differences in intestinal cell surface and epithelial cell integrity [20], especially they are very sensitive to the animal species because of differences in physiological conditions [21]. More importantly, those factors can make substantial contribution to data inhomogeneity that, in turn, can create paramount obstacles to producing a sound quantitative theoretical model based on the data compiled from the public domain since only homogenous data can produce a good in silico model [22].

In silico technologies have been seamlessly integrated into the drug discovery and development and they especially provide valuable advantages in ADME/Tox profiling due to their extremely fast throughput and low cost [23]. As such, it is plausible to expect an in silico model that can predict intestinal permeability is very useful. Nevertheless, no sound quantitative structure–activity relationship (QSAR) model has been published to date despite, even though some qualitative studies have been conducted. The scarcity in QSAR model can be plausibly attributed to the lack of consistent and homogenous data in the public domain and, more importantly, the extremely complex process of intestinal permeability (vide supra) since it can take place through various active transport and passive diffusion routes. More specifically, the SLC transporters can enhance the drug uptake into the intestine and hence increase drug absorption, whereas ABC proteins can elevate drug efflux out of intestine and therefore reduce drug absorption [24], leading to problematic situations for model development. For instance, the substrates of PepT1 and P-gp, which are two of the most abundant SLC and ABC transporters, respectively, in jejunum [15], can interact with their transporter proteins by hydrogen-bond donor (HBD) [25,26], suggesting that HBD can simultaneously promote and hinder intestinal permeability. As such, traditional or machine learning (ML) modeling schemes are not sophisticated enough to manage such exceedingly nonlinear situations.

Accordingly, it is extraordinarily difficult, if not entirely infeasible, to develop a robust in silico model to predict the intestinal permeability with the consideration of those critical factors governing the perplexing efflux and influx active transport and passive diffusion mentioned above. Such challenge, nevertheless, can be solved by the novel ML-based hierarchical support vector regression (HSVR) scheme devised by Leong et al. [27] since HSVR can properly depict the complicated and inconstant dependencies of descriptors that can be greatly attributed to the fact that HSVR has the advantageous features of both a local model and a global model, namely larger coverage of applicability domain (AD) and a higher degree of predictivity, respectively. Unlike most predictive models, which are vulnerable to the “mesa effect”, i.e., give mediocre performances when applied to extrapolated predictions, HSVR can substantially minimize such performance deterioration, as demonstrated elsewhere [1,28,29], suggesting that HSVR is insusceptible to outliers in contrast to the other predictive models that is of crucial importance to a theoretical model [30]. Herein, this investigation was aimed at developing an accurate, rapid, and predictive in silico model based on the HSVR scheme to predict the intestinal permeability to facilitate drug discovery and development.

## 2. Results

### 2.1. Data Partition

**Figure 1:**
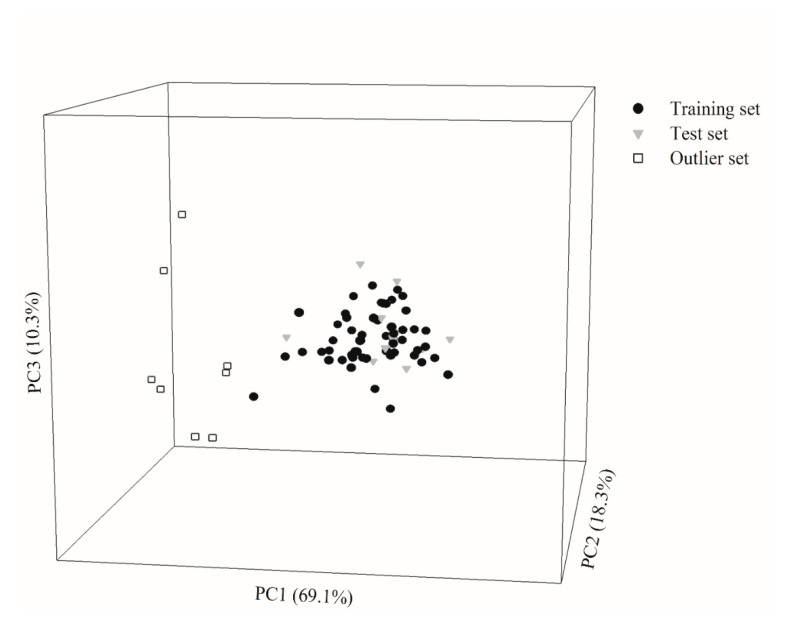
Molecule distribution for the molecules enrolled in this investigation in the training set (solid circle), test set (gray triangle), and outlier set (open square) in the chemical space spanned by three principal components.

Of all molecules selected in this study, 53 and 13 molecules were randomly assigned to the training set and test set, respectively, with a ca. 4:1 ratio. Figure 1 shows the projection of all molecules enrolled in this investigation in chemical space, spanned by the first three principal components (PCs), which characterized 97.7% of the total variable variance. It can be observed that the training samples and test samples showed similar distributions in the chemical space. Furthermore, the high degrees of biological and chemical similarity between both datasets can also be observed in Figure S1, which displays the histograms of log *P*_eff_, molecular weight (MW), log *D*, log *P*, hydrogen-bond acceptor (HBA), hydrogen-bond donor (HBD), and polar surface area (PSA) in density form for the training and test molecules. Thus, the unbiased partition of data samples can be ascertained [31].

It is not trivial to establish the AD of predictive models prior to model development to identify the outliers and exclude them from data collection [32]. There are various versions to define AD [33]. This study adopted the version based on the chemical similarity/dissimilarity using principal component analysis (PCA) to graphically assess the outliers [32]. Those designated outliers, conversely, are very dissimilar from the training samples, as manifested by the fact that they are totally situated outside the perimeter of the training set in the chemical space shown in Figure 1. In fact, the differences between the outliers and the other samples can be realized by the fact that their surface areas are unanimously more than 600 Å^2^, whereas surface areas of the others are less than 600 Å^2^.

### 2.2. SVRE

Various SVR models were built based on various descriptor combinations and runtime parameters, and three SVR models, denoted as SVR A, SVR B, and SVR C, were selected to construct the SVR ensemble, which, in turn, was subjected to regression by another SVR to generate the HSVR model. The optimal runtime parameters of SVR A, SVR B, SVR C, and HSVR, are listed in Table S2.

These three SVR models, which unanimously selected four descriptors with different combinations (Table 1), were adopted based on their individual performances on the molecules and statistical assessments in the training set and test set. Table S1 lists their predictive log *P*_eff_ values. Table 2 and Table 3 summarize the corresponding statistical assessments of these three SVR models in the training set and test set, respectively.

**Figure 2:**
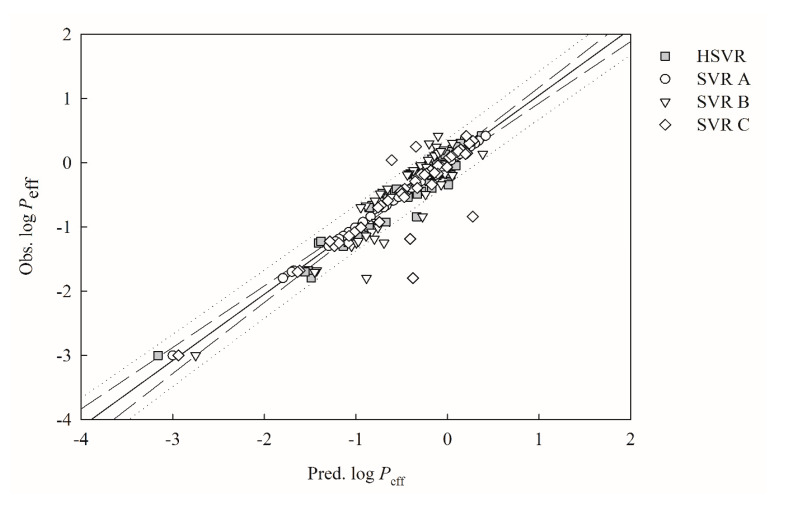
Observed log *P*_eff_ vs. the log *P*_eff_ predicted by SVR A (open circle), SVR B (open triangle), HSVR SVR C (open diamond), and HSVR (gray square) for the molecules in the training set. The solid line, dashed lines, and dotted lines correspond to the HSVR regression of the data, 95% confidence intervals for the HSVR regression, and 95% confidence interval for the prediction, respectively.

Figure 2 displays scatter the plot of observed versus the predicted log *P*_eff_ values by SVR A, SVR B, SVR C, and HSVR for the molecules in the training set. The predictions by SVR A, SVR B, and SVR C are generally in good agreement with the observed values for most of the molecules in the training set, as depicted by their small MAE and *s* values (Table 2). Furthermore, Figure 2 shows that most of the points predicted by SVR B mostly lie on or are closer to the regression line when compared with SVR A and SVR C. As such, SVR B generated the lowest Δ_Max_ (0.91), MAE (0.25), *s* (0.15), and RMSE (0.29) and the largest *r*^2^ parameter (0.83), suggesting that SVR B executed better than SVR A and SVR C for the molecules in the training set. Nevertheless, SVR B produced not only the lowest $q_{\mathrm{CV}}^{2}$ value (0.02) but also the largest difference between *r*^2^ and $q_{\mathrm{CV}}^{2}$ (0.81) when subjected to the leave-one-out cross-validation (Table 2), signifying its high level of overtraining that, in turn, can severely limit its practical application. SVR A, SVR B, and SVR C unanimously gave rise to the miniature <$r_{s}^{2}$> values of 0.02 (Table 2) when subjected to the *Y*-scrambling, and their almost zero values of <$r_{s}^{2}$> apparently depict that there is little chance correlation associated with those SVR models [34].

**Figure 3:**
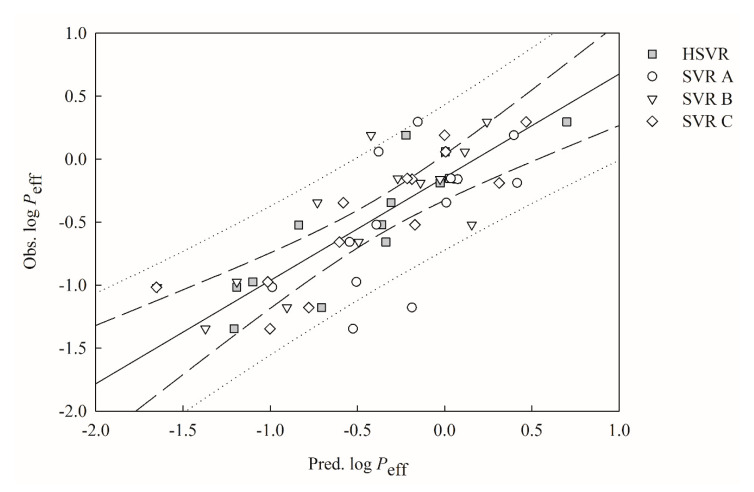
Observed log *P*_eff_ vs. the log *P*_eff_ predicted by SVR A (open circle), SVR B (open triangle), HSVR SVR C (open diamond), and HSVR (gray square) for the molecules in the test set. The solid line, dashed lines, and dotted lines correspond to the HSVR regression of the data, 95% confidence intervals for the HSVR regression, and 95% confidence interval for the prediction, respectively.

The predictions by SVR A, SVR B, and SVR C in the test set are in modest agreement with the experimental values, as shown in Figure 3, which displays scatter the plot of observed versus the predicted log *P*_eff_ values by SVR A, SVR B, SVR C, and HSVR for the molecules in the test set. Nevertheless, the mean absolute errors calculated by SVR A, SVR B, and SVR C increase from 0.33, 0.25, and 0.15 in the training set to 0.39, 0.26, and 0.24 in the test set, respectively (Table 3). The other statistical assessments also indicate the performance deteriorations of these three models in the SVRE from the training set to the test set (Table 2 and Table 3).

Furthermore, SVR A, SVR B, and SVR C yielded *q*^2^ values of 0.40, 0.67, and 0.73 in the test set, which are smaller than their *r*^2^ counterparts in the training set. Most notably, the difference between *r*^2^ and *q*^2^ evaluated by SVR A was 0.22, indicating the overtraining nature of SVR A that is also confirmed by its $q_{F1}^{2}$, $q_{F2}^{2}$, $q_{F3}^{2}$, and *CCC*.

**Figure 4:**
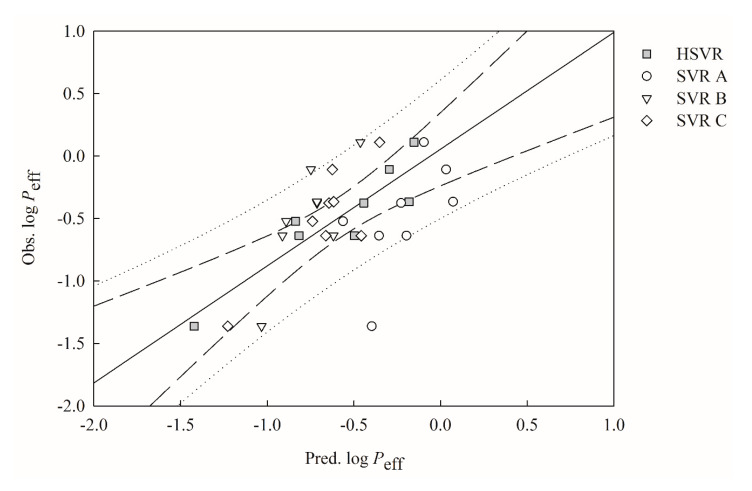
Observed log *P*_eff_ vs. the log *P*_eff_ predicted by SVR A (open circle), SVR B (open triangle), HSVR SVR C (open diamond), and HSVR (gray square) for the molecules in the outlier set. The solid line, dashed lines, and dotted lines correspond to the HSVR regression of the data, 95% confidence intervals for the HSVR regression, and 95% confidence interval for the prediction, respectively.

Significant performance decreases can be observed when those SVR models in the ensemble were applied to the molecules in the outlier set, as depicted by the statistical parameters listed in Table 4. SVR A, SVR B, and SVR C, for instance, gave rise to $q_{F1}^{2}$ values of −0.10, 0.04, and 0.47, respectively, which differ greatly from their *r*^2^ values in the training set (Table 2). In addition, they showed larger distances between points and the regression line in the outlier set when compared with their counterparts in the training set, as displayed in Figure 4. Such substantial variations in performance can be realized by the fact most predictive models are vulnerable to the “mesa effect”, which leads to a predictive model executing poorly when applied to extrapolated predictions [35].

### 2.3. HSVR

The HSVR model was yielded by the regression of the SVR ensemble based on the predictions of all samples and statistical evaluations in the training set (Table S1 and Table 2) and its optimal runtime conditions are listed in Table S2. HSVR generally executed better than SVR A, SVR B, and SVR C for those training samples, as illustrated by Figure 2, in which it can be observed that the distances between the predictions by HSVR and regression line generally fall in the range between the largest ones and smallest ones produced by its SVR counterparts in the ensemble. Nevertheless, HSVR predicted better than all of the models in the SVRE in some cases as demonstrated by the prediction of compound **2** (aloin), in which SVR A, SVR B, SVR C, and HSVR yielded absolute residuals of 0.25, 0.20, 0.06, and 0.03, respectively. As such, HSVR statistically functioned better than SVR A, SVR B, and SVR C, as manifested by all parameters listed in Table 2 except MAE values, which were 0.15 and 0.16 for SVR C and HSVR, respectively. In addition, HSVR gave rise to the largest *r*^2^ value (0.93) as compared with its counterparts in the SVRE. In addition, there is a little chance that HSVR was produced by chance correlation, as manifested by its nearly zero value of <$r_{s}^{2}$> (0.02) [34].

When applied to the molecules in the test set, slight performance decreases can be observed for HSVR. For instance, RMSE increased from 0.17 in the training set to 0.26 in the test set (Table 2 and Table 3). Nevertheless, the parameter Δ_Max_ declined from 0.50 in the training set to 0.47 in the test set. Figure 3 shows that HSVR performed better than SVR A, SVR B, and SVR C in the test set. The performance dominance of HSVR can be further confirmed by those statistical evaluations listed in Table 3. For example, SVR A, SVR B, SVR C, and HSVR generated *s* values of 0.29, 0.24, 0.20, and 0.14, respectively. Similar observation that HSVR gave rise to smaller absolute residuals than its counterparts in the SVRE can also be noted in the test set. The absolute prediction deviation of compound **59**, for instance, was 0.04 yielded by HSVR, whereas SVR A, SVR B, and SVR C gave rise to the absolute residuals of 0.35, 0.38, and 0.24, respectively. HSVR normally generated consistent and small errors in both training and test sets, as depicted by those parameters listed in Table 2 and Table 3, when compared with its SVR counterparts in the ensemble. Moreover, HSVR yielded the largest *q*^2^ (0.81) in the test set and the smallest difference between *r*^2^ and $q_{\mathrm{CV}}^{2}$ (0.09), suggesting that HSVR was well-trained or no overfitting effect was observed because it would otherwise generate a significant difference between *r*^2^ and *q*^2^ or between *r*^2^ and $q_{\mathrm{CV}}^{2}$.

When applied to the outliers, HSVR even showed more pounced predominance, as indicated by those statistical parameters listed in Table 5, from which it can be recognized that HSVR generated the largest *q*^2^ values and smallest deviation-related parameters. The superiority of HSVR in the outlier set can be plausibly due to the broad applicability domain encompassed by HSVR as compared with its SVR counterparts in the ensemble and, more importantly, the more robust nature of HSVR makes it more practically useful in real applications [30].

### 2.4. Predictive Evaluations

**Figure 5:**
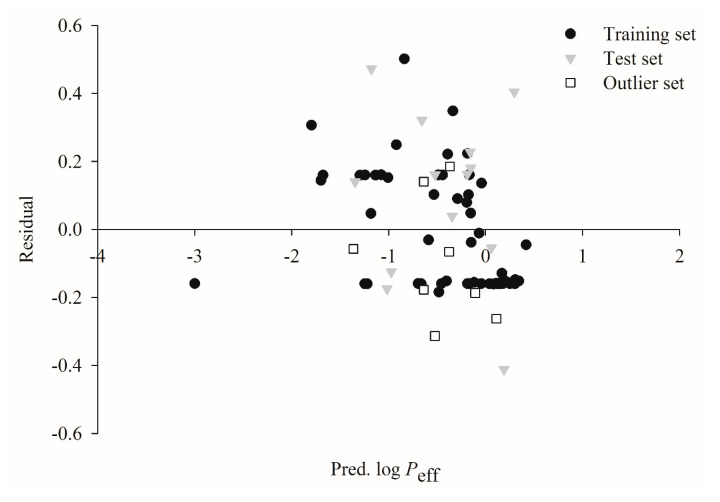
Residual vs. the log *P*_eff_ predicted by HSVR in the training set (solid circle), test set (gray triangle), and outlier set (open square).

Figure 5 illustrates the scatter plots of the residual vs. the log *P*_eff_ values predicted by HSVR for the molecules in the training set, test set, and outlier set. It can be conceived that the residuals are approximately evenly allocated on both sides of *x*-axis along the range of predicted values in all datasets, suggesting that there is no significant systematic error associated with HSVR. The unbiased predictions can be further rendered by its almost negligible average residuals that were 0.00, −0.10, and 0.09 in the training set, test set, and outlier set, respectively (Table S1).

The derived HSVR model was further assessed by combining the most rigorous validation criteria collectively suggested by Golbraikh et al. [36], Ojha et al. [37], Roy et al. [38], and Chirico and Gramatica [39] in the training set, test set, and outlier set (Equations (16)–(22)). The results are listed in Table 5, from which it can be found that HSVR showed similar high levels of performance in those three datasets. More importantly, HSVR completely fulfilled all validation requirements, suggesting that this predictive model is highly accurate and predictive.

### 2.5. Mock Test

**Figure 6:**
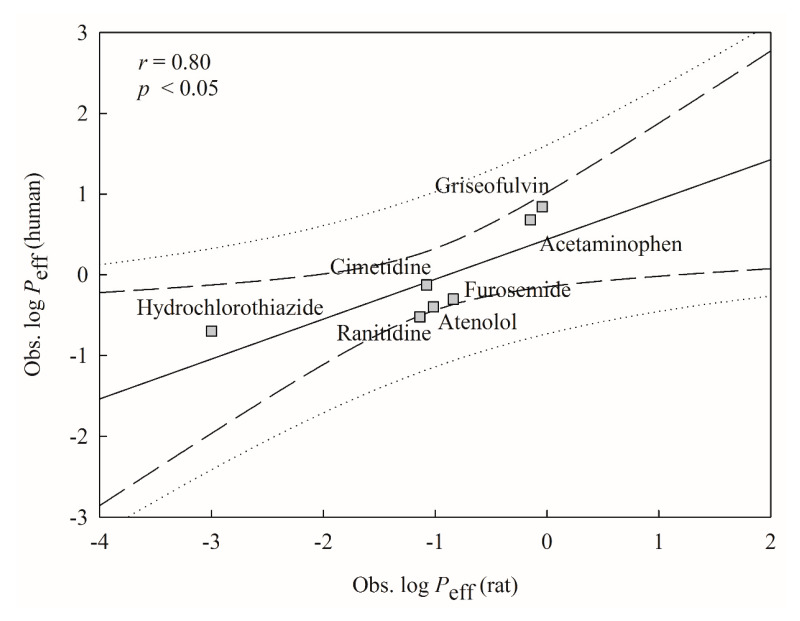
Observed human log *P*_eff_ versus observed rat log *P*_eff_ for the molecules in the mock test. The solid line, dashed lines, and dotted lines correspond to the mock test regression of the observed data, 95% confidence interval for the mock test regression, and 95% confidence interval for the observation, respectively.

To imitate real world challenges, the derived HSVR model was checked by all marketed drugs assayed by Lennernäs [40], of which seven were also included in this investigation, producing a good way to calibrate the challenging system. However, Lennernäs measured the *P*_eff_ values by the SPIP in human jejunum in contrast to the compounds enrolled in this study, whose *P*_eff_ values were obtained using the rat jejunum segment. Thus, those drugs assayed by Lennernäs are not suitable as the test set or second external set since their validation assessments (vide supra) are not applicable to these drugs. The subsequent relationship between both measured systems was initially instituted and checked based on those common seven drugs and the resulted scattered plot is displayed in Figure 6. The results show that both systems were modestly correlated with each other with an *r* value of 0.80, suggesting that it is plausible to validate the derived HSVR model by those novel molecules assayed by Lennernäs, which is consistent with the fact that the rat SPIP *P*_eff_ values can be useful to predict human intestinal permeability [17].

**Figure 7:**
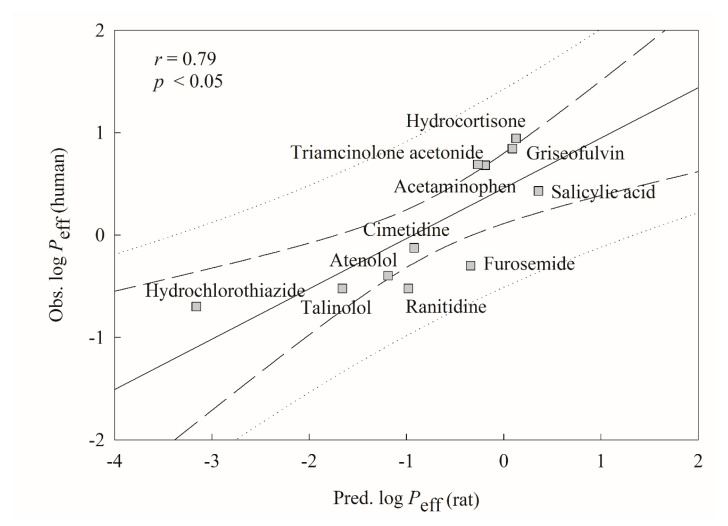
Observed human log *P*_eff_ versus the rat log *P*_eff_ predicted by HSVR for the molecules in the mock test. The solid line, dashed lines, and dotted lines correspond to the HSVR regression of the data, 95% confidence interval for the HSVR regression, and 95% confidence interval for the prediction, respectively.

Figure 7 displays the tested results of 11 novel drugs. It can be observed that the *r* value between experimental human log *P*_eff_ and predicted rat log *P*_eff_ was 0.79. The negligible difference between both numbers (0.80 vs. 0.79) suggests that HSVR can almost reproduce the experimental observations. Accordingly, this mock challenge by 11 marketed drugs apparently assured the predictivity of HSVR and it is plausible to adopt this HSVR model as a surrogate for preliminary estimation of human intestine permeability in the process of drug discovery and development.

## 3. Discussion

Numerous in silico models have been reported to predict intestinal permeability [41,42,43,44,45,46,47,48,49,50,51,52,53,54,55]. However, those published models were based on data assayed by different experimental conditions or different measured parameters, and some of them were qualitative models, making the direct comparison between HSVR and those models extremely difficult. In addition, intestine permeability is an extremely complicated process, which can take place through various active transport and passive diffusion routes (vide supra). As such, it is not uncommon to observe that various descriptor combinations associated with intestinal permeability have been identified. For example, Shultz proposed the significance of HBD, topological polar surface area (TPSA), and log *P* in intestinal permeability [56], whereas Broccatelli et al. recognized the contributions of TPSA, MW, HBD, number of rotamers (*n*_rot_), charge, and fraction ionized at pH 7.4 (cFI_7.4_) to intestinal permeability [57].

Drugs must pass through the hydrophobic mucus layer, which is adjacent to the intestinal wall, before they can penetrate through the intestinal epithelial cells [58]. As such, hydrophobicity is of critical importance in intestinal permeability and it can be represented by the *n*-octanol–water partition coefficient (log *P*) and the *n*-octanol–water partition coefficient at pH 6.5 (log *D*). Moreover, it was proposed by Balimane et al. that log *P* and log *D* should be adopted to predict the intestinal permeability since log *P* alone is not sufficient enough to accurately render this complicated process [9]. As such, both log *P* and log *D* were adopted by this study (Table 1). However, the selection of both descriptors can plausibly lead to an overtrained model since the correlation coefficient between log *P* and log *D* was 0.73 for all molecules included in this study. This controversial issue can be eliminated by the fact that log *D* was adopted by SVR A and SVR B, whereas log *P* was selected by SVR C, depicting the fact that no single SVR model included both descriptors simultaneously. In fact, this dilemma of selecting both correlated descriptors to accurately predict intestinal permeability cannot be resolved by any other traditional linear or machine learning-based QSAR schemes but only by any ensemble-based scheme such as HSVR.

It has been observed that PSA is profoundly implicated in membrane permeability in passive diffusion [59], which is completely consistent with the PAMPA study [1] as well as intestinal permeability [56]. In addition, permeability also relies on MW, as proposed in [13]. Nevertheless, neither PSA nor MW was adopted by any of the SVR models in the ensemble (Table 1). Conversely, it is seemingly unusual to observe that the descriptor *n*_N+O_ was selected by SVR A and yet has hitherto not been adopted by any reported study. These discrepancies can be realized by the fact that *n*_N+O_ was modestly correlated with PSA and MW with *r* values of 0.88 and 0.71, respectively, for all molecules selected in this study. The empirical observation indicated that models with the selection of *n*_N+O_ performed better than those with the selection of PSA or MW (data not shown), plausibly due to the descriptor–descriptor interaction [1], suggesting that it is plausible to represent PSA or MW by *n*_N+O_. The negative correlation between *n*_N+O_ and log *P*_eff_ (−0.29) is also consistent with the fact that permeability can decrease with MW [60].

**Figure 8:**
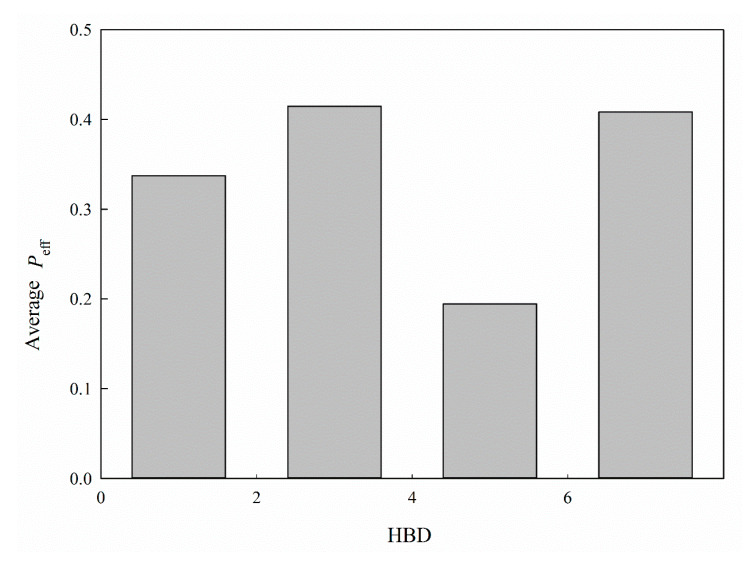
Average *P*_eff_ vs. the distribution of HBD.

It has been postulated that hydrogen bond, which can be characterized by HBA and HBD, plays a critical role in intestinal P-gp-mediated transport [61] and HBD makes substantial contributions to intestinal permeability when compared with its HBA counterpart [56]. Accordingly, HBD was adopted in this study. Nevertheless, the relationship between HBD and *P*_eff_ is seemingly obscure, as illustrated by Figure 8, which shows the average *P*_eff_ for each histogram bin of HBD for all molecules included in this investigation. This peculiar relationship can be plausibly attributed to the fact that the substrates of PepT1 and P-gp, which are the most abundant SLC and ABC transporters, respectively, in jejunum [15], can interact with their transporter proteins via HBD [25,26]. The complexity can be further increased by taking into the account the fact that P-gp inhibitors, modulators, and substrates can interact with P-gp through HBD [26,62,63]. As such, HBD can simultaneously facilitate and hinder intestinal permeability, leading to a perplexing dependency, which, in turn, can create an unsurmountable hurdle for creating a predictive theoretical model regardless of traditional linear or machine learning-based schemes.

**Figure 9:**
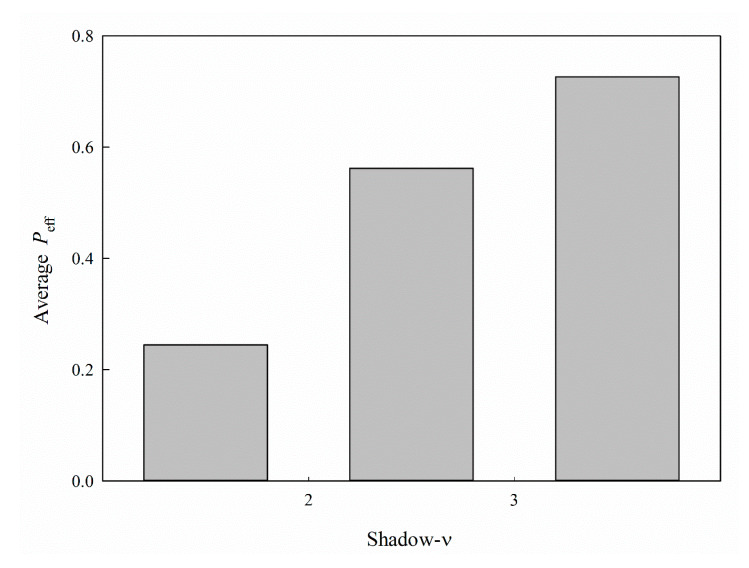
Average *P*_eff_ vs. the distribution of shadow-*ν*.

Shadow-*ν* is a size-related descriptor which measures the ratio of largest to smallest dimension. It can be observed in Figure 9, which displays the average *P*_eff_ for each histogram bin of shadow-*ν*, that *P*_eff_ generally increased with shadow-*ν* for all molecules selected in this investigation, suggesting that molecules with larger shadow-*ν* have higher permeability than their smaller counterparts.

**Figure 10:**
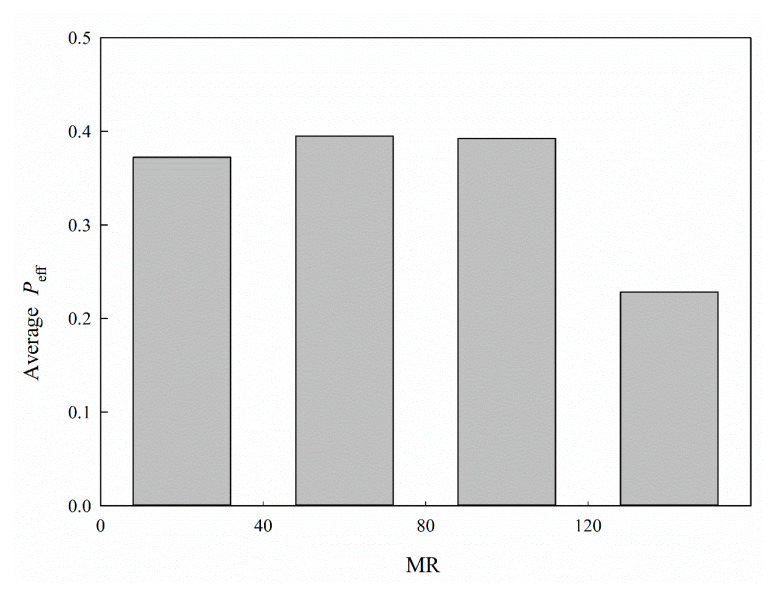
Average *P*_eff_ vs. the distribution of MR.

It has been observed that molar refractivity (MR), which is possibly associated with molecular size, polarity, and/or polarizability [64], is closely related to ligand‒P-gp interactions [65,66]. Nevertheless, little correlation manifested between MR and log *P*_eff_ for all molecules enrolled in this study, with an insignificant *r* value of −0.12 (Table 1). This incongruity can be resolved by the nonlinearity between MR and *P*_eff_, as demonstrated in Figure 10, which illustrates the average *P*_eff_ for each histogram bin of MR. It can be observed that *P*_eff_ marginally increased with MR and substantially decreased afterwards, suggesting the nonlinear relationship between MR and *P*_eff_. Thus, linear models cannot properly render such a complicated relationship.

The significance of the descriptor *μ* in intestinal permeability has been recognized [67] since *μ* can describe the solute‒solute and solute–solvent dipole interactions [68], as demonstrated in PAMPA permeability [1], leading to nonlinear relationship between *μ* and permeability. In addition, it has been observed that ligands can interact with the efflux transporter P-gp and the influx transporter PepT1 through dipole interactions [69,70,71], giving rise to the complex role played by *μ* in intestinal permeability.

It is of interest that most of descriptors selected in this study are associated with passive diffusion, which is consistent with the fact that passive diffusion is the major route for intestinal permeability for many administrated drugs [12]. Additionally, MR, shadow-*ν*, and *n*_N+O_, which was selected in place of MW in this study (vide supra), are closely linked to molecular size, and the molecular size is a determining barrier factor in intestinal permeability as postulated [72,73,74].

**Figure 11:**
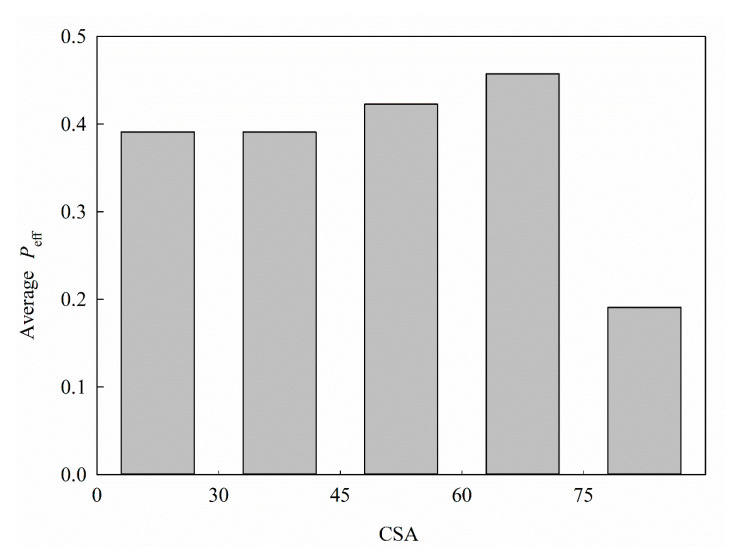
Average *P*_eff_ vs. the distribution of CSA.

CSA, which is also another characteristic associated with molecular size, has been implicated in membrane permeability [75]. Figure 11 exhibits the average *P*_eff_ for each histogram bin of CSA. It can be observed that *P*_eff_ did not show substantial variations with CSA initially, yet the *P*_eff_ value greatly dropped once CSA was larger than 75, which is very similar to the trend observed for MR (Figure 10), suggesting that it is more difficult to penetrate the intestinal wall once the CSA values are larger than a threshold. Nevertheless, the empirical observation has indicated that HSVR with the selections of MR executed better than those with the selection of CSA (data not shown), presumably because of the descriptor–descriptor interaction [1].

**Figure 12:**
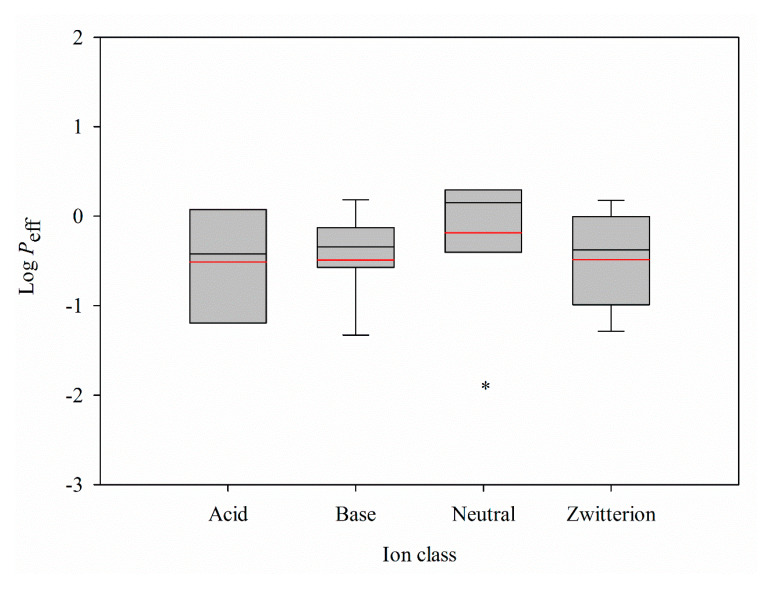
Box plot of log *P*_eff_ values for different ion classes, where the boxes indicate the distribution of log *P*_eff_ from the 25th to the 75th percentile, the black and red lines represent the median and mean values, the whiskers depict the minimum and maximum values, and the asterisk denotes significant difference between neutral and the others (*p* < 0.05).

It has been indicated that ion class is one of critical factors in physiological-based pharmacokinetic (PBPK) models and ADME/Tox properties that should be taken into account [20,76]. Actually, it has been demonstrated that neutral compounds show higher passive diffusion [1]. Accordingly, all molecules enrolled in this investigation were subjected to ion class analysis. Figure 12 displays the box plot of the log *P*_eff_ minimum, maximum, mean, median, the 25th percentile, and the 75th percentile for each ion class. The log *P*_eff_ values of neutral compounds are statistically greater than the other ion classes, depicting that neutral compounds show higher intestinal permeability. It is possible to improve the compound’s intestinal permeability of the other ion classes by chemical modification to produce neutral compounds when they show low intestinal permeability.

Initially, massive attempts were made in this investigation to construct various 2-QSAR models by adopting numerous partial least squares (PLSs), but no acceptable models were yielded (data not shown) [29]. This challenge was due to little correlation between the selected descriptors and log *P*_eff_ for those molecules selected in this study and the largest absolute maximum *r* was merely 0.29 between *n*_N+O_ and log *P*_eff_ (Table 1), depicting the highly nonlinear relationship between them. More importantly, the substantial difference in 2-QSAR development between the passive diffusion, viz. the PAMPA system, and intestinal permeability can be greatly attributed to the significant and complex roles played by those active (influx and efflux) transporters. As such, it is extremely difficult to build a sound linear model to predict intestinal permeability. Conversely, the accurate and predictive HSVR model can considerably delineate such nonlinear dependence of log *P*_eff_ on descriptors.

## 4. Materials and Methods 

### 4.1. Data Compilation

A comprehensive literature search was carried out to retrieve in vivo permeability data from a variety of sources to construct quality data for this investigation. Of various assay systems, 74 compounds, which were measured by SPIP in rat jejunum with pH 6.5 phosphate buffered saline (PBS), were adopted from various sources [77,78,79,80,81,82,83,84,85,86,87,88,89,90,91,92,93,94,95,96,97,98]. The mean value was taken to assure better consistency if there were more than one *P*_eff_ values for a given compound within close range. Chemical structures without defined stereochemistry (e.g., racemates) were discarded from the collection. All molecules included in this study, IUPAC names, SMILES strings, CAS registry numbers, logarithm of observed *P*_eff_ values, and references to the literature are listed in Table S1.

### 4.2. Descriptor Enumeration

All molecules selected in this study were subjected to full geometry optimization using the density functional theory (DFT) B3LYP method with the basis set 6-31G(*d*,*p*) by the *Gaussian* package (Gaussian, Wallingford, CT) since it has been demonstrated elsewhere that predictive models with the selection of DFT descriptors can perform better [29]. To mimic the assay conditions, the water solvent system was considered by the polarizable continuum model (PCM) [99,100]. The minimum of optimized geometry on the potential energy surface was verified by force calculations in case no imaginary frequency was obtained. Furthermore, atomic charges were also determined by the molecular electrostatic potential-based method of Merz and Kollman [101]. The highest occupied molecular orbital energy (*E*_HOMO_), lowest unoccupied molecular orbital energy (*E*_LUMO_), free energy (Δ*G*), molecular dipole (*μ*), and its maximum absolute components (|*μ|*_Max_) of each molecule were also retrieved from the optimization calculations.

More than 200 one-, two-, and three-dimensional molecular descriptors, which can be categorized as electronic descriptors, spatial descriptors, structural descriptors, thermodynamic descriptors, topological descriptors, and *E*-state indices, were evaluated by the *Discovery Studio* package (BIOVIA, San Diego, CA, USA) and *E-Dragon* (available at the website: http://www.vcclab.org/lab/edragon/). Additionally, the cross-sectional area (CSA) was also computed using the method modified by Muehlbacher et al. since it was implicated in membrane permeability [102]. Molecules were further placed into four classes, namely neutral, zwitterion, acid, and base, by their p*K*_a_ values. Specifically, molecules with only one p*K*_a_ value are defined as neutrals, whereas those with more than one p*K*_a_ value are designated as zwitterions, acids, and bases when their largest p*K*_a_ values are larger than 7 and the smallest p*K*_a_ values are smaller than 7; the largest and smallest p*K*_a_ values are smaller than 7; and the largest and smallest p*K*_a_ values are larger than 7, respectively.

Descriptor filtration was initiated by discarding those descriptors missing for at least one sample or barely displaying discrimination against most of samples. It was suggested by Topliss and Edwards that the probability of spurious correlations can be reduced by removing those descriptors with intercorrelation values of *r*^2^ > 0.80 [103]. Accordingly, the Spearman’s matrix was constructed and a more conservative threshold of *r*^2^ ≧ 0.64 was adopted. Descriptor normalization, which was designated to reduce the possibility of descriptors with broader ranges outweighing those with narrower ranges, was implemented by

(3) $$\chi_{ij}=\left(x_{ij}-\langle x_{j}\rangle \right)/{\left[\sum_{i=1}^{n}{\left(x_{ij}-\langle x_{j}\rangle \right)}^{2}/\left(n-1\right)\right]}^{1/2}$$

where $x_{ij}$ and $\chi_{ij}$ present the *j*th original and normalized descriptors of the *i*th sample, respectively; $\langle x_{j}\rangle$ is the mean value of the original *j*th descriptor; and *n* is the number of samples.

Descriptors are one of the critical factors influencing the performance of predictive models [104]. The initial descriptor selection was executed by genetic function approximation (GFA) using the QSAR module of *Discovery Studio* due to its effectiveness and efficiency [105]. Further selection was done by the recursive feature elimination (RFE) scheme, in which the model was repeatedly built by all but one of descriptors. Their contributions to the predictive performance were then evaluated, and the descriptor with the smallest contribution was discarded [106].

### 4.3. Sample Partition

It is of necessity to identify the outliers prior to the model development, which can be done by examining the molecular distribution in the chemical space [107]. As such, the chemical space was initially constructed based on principal components (PCs) using the Diverse Molecules/Principal Component Analysis module of *Discovery Studio* and the outliers were determined. The remaining compounds were randomly divided into the training set and test set with a ca. 4:1 ratio as proposed to build and validate the models, respectively [108], using the Diverse Molecules/Library Analysis module of *Discovery Studio*. In addition, it was suggested by Golbraikh et al. that only high levels of chemical and biological similarity between the training samples and test samples can develop a sound model [36]. As such, the data distribution was carefully examined to guarantee the high levels of biological and chemical similarity in both datasets.

### 4.4. Hierarchical Support Vector Regression

Hierarchical support vector regression (HSVR), which was originally proposed by Leong et al. [27], was evolved from support vector machine (SVM), which was originally invented by Vapnik et al. [109]. Initially, SVM was devised for classification and then modified for regression, termed support vector regression (SVR) [110]. The most distinguished difference between SVR and HSVR is that the latter can simultaneously take into account the characteristics of a global model, viz. broader applicability domain (AD), and a local model, viz. higher predictivity, as compared with the former [28]. More importantly, it has been demonstrated by a number of studies that predictive models based on the HSVR scheme perform extremely well [1,26,27,28].

The principle and implementation of HSVR has been detailed elsewhere and the architecture of HSVR schemeis depicted in Figure 1 of Leong et al. [27]. Briefly, SVR models were developed by the *svm-train* module in *LIBSVM* (software available at: http://www.csie.ntu.edu.tw/~cjlin/libsvm) using those samples in the training set with various descriptor combinations and SVR run parameters. The derived models were validated by *svm-predict* using those samples in the test set. The regression functions, namely *ε*-SVR and *γ*-SVR, were tried, and radial basis function (RBF) was selected as the kernel function due to its simplicity and greater performance [111]. The runtime parameters including regression modes *ε*-SVR and *ν*-SVR, the corresponding *ε* and *ν*, cost *C*, and the kernel width *γ* were automatically run by the systemic grid search algorithm in a parallel fashion.

The principle of Occam’s razor was applied to the construction of SVR ensemble (SVRE), which suggests that the fewer the ensemble members, the better [112]. More specifically, two SVR models were selected to develop an SVRE, which was further subjected to regression by another SVR to give rise to the final HSVR model. The construction of two-member SVREs was repeated until the HSVR model executed well. Otherwise, three- or even four-member ensembles would be built by adding one or more SVR models, respectively, in the case all two-member ensembles failed to show good performance.

### 4.5. Predictive Assessment

The difference between the observed value ($y_{i}$) and the predicted value (${\hat{y}}_{i}$), viz. the residual, was calculated

(4) $$\Delta_{i}=y_{i}-{\hat{y}}_{i}$$

The root mean square error (RMSE) and the mean absolute error (MAE) for *n* samples in the dataset were computed

(5) $$\mathrm{RMSE}={\left[\sum_{i=1}^{n}\Delta_{i}^{2}/n\right]}^{1/2}$$

(6) $$\mathrm{MAE}=\frac{1}{n}\sum_{i=1}^{n}\left|\Delta_{i}\right|$$

The developed models were further assessed by the correlation coefficients *r*^2^ and *q*^2^ in the training set and external set, respectively, by the following equation

(7) $$r^{2},\;q^{2}=1-\sum_{i=1}^{n}{\left({\hat{y}}_{i}-y_{i}\right)}^{2}/\sum_{i=1}^{n}{\left(y_{i}-\langle \hat{y}\rangle \right)}^{2}$$

where $\langle \hat{y}\rangle$ represents the average predicted value.

Additionally, Ojha et al. [37] also proposed various modified versions of *r*^2^ to gauge the model performance

(8) $$r_{m}^{2}=r^{2}\left(1-\sqrt{\left|r^{2}-r_{o}^{2}\right|}\right)$$

(9) $${r^{\prime }}_{m}^{2}=r^{2}\left(1-\sqrt{\left|r^{2}-{r^{\prime }}_{o}^{2}\right|}\right)$$

(10) $$\langle r_{m}^{2}\rangle =\left(r_{m}^{2}+{r^{\prime }}_{m}^{2}\right)/2$$

(11) $$\Delta r_{m}^{2}=\left|r_{m}^{2}-{r^{\prime }}_{m}^{2}\right|$$

where the correlation coefficient $r_{o}^{2}$ and the slope *k* were derived from the regression line (predicted vs. observed values) without the intercept, whereas ${r^{\prime }}_{o}^{2}$ was calculated from the regression line (observed vs. predicted values) without the intercept.

The developed model was further subjected to internal validation using the leave-one-out cross-validation scheme, producing the correlation coefficient $q_{\mathrm{CV}}^{2}$. Furthermore, *Y*-scrambling or *Y*-randomization was applied by randomly permuting the log *P*_eff_ values, viz. *Y* values, to refit the previously derived models while the descriptors remained unchanged. This procedure was repeated 25 times, as suggested in [34], to give rise the average correlation coefficient $\langle r_{s}^{2}\rangle$ to ensure no chance correlation was associated with those derived models.

Additionally, the predictivity in the external dataset was evaluated by the correlation coefficients $q_{F1}^{2}$, $q_{F2}^{2}$, and $q_{F3}^{2}$, and concordance correlation coefficient (*CCC*) using *QSARINS* [32,113]

(12) $$q_{F1}^{2}=1-\sum_{i=1}^{n_{\mathrm{EXT}}}{\left(y_{i}-{\hat{y}}_{i}\right)}^{2}/\sum_{i=1}^{n_{\mathrm{EXT}}}{\left(y_{i}-\langle y_{\mathrm{TR}}\rangle \right)}^{2}$$

(13) $$q_{F2}^{2}=1-\sum_{i=1}^{n_{\mathrm{EXT}}}{\left(y_{i}-{\hat{y}}_{i}\right)}^{2}/\sum_{i=1}^{n_{\mathrm{EXT}}}{\left(y_{i}-\langle y_{\mathrm{EXT}}\rangle \right)}^{2}$$

(14) $$q_{F3}^{2}=1-\left[\sum_{i=1}^{n_{\mathrm{EXT}}}{\left(y_{i}-{\hat{y}}_{i}\right)}^{2}/n_{\mathrm{EXT}}\right]/\left[\sum_{i=1}^{n_{\mathrm{TR}}}{\left(y_{i}-\langle y_{\mathrm{TR}}\rangle \right)}^{2}/n_{\mathrm{TR}}\right]$$

(15) $$CCC=\frac{2\sum_{i=1}^{n_{\mathrm{EXT}}}\left(y_{i}-\langle y_{\mathrm{EXT}}\rangle \right)\left({\hat{y}}_{i}-\langle {\hat{y}}_{\mathrm{EXT}}\rangle \right)}{\sum_{i=1}^{n_{\mathrm{EXT}}}{\left(y_{i}-\langle y_{\mathrm{EXT}}\rangle \right)}^{2}+{\left({\hat{y}}_{i}-\langle {\hat{y}}_{\mathrm{EXT}}\rangle \right)}^{2}+n_{\mathrm{EXT}}{\left(\langle y_{\mathrm{EXT}}\rangle -\langle {\hat{y}}_{\mathrm{EXT}}\rangle \right)}^{2}}$$

where *n*_TR_ and *n*_EXT_ are the numbers of samples in the training set and external set, respectively; $\langle {\hat{y}}_{\mathrm{TR}}\rangle$ stands for the average predicted value in the training set; and $\langle y_{\mathrm{EXT}}\rangle$ and $\langle {\hat{y}}_{\mathrm{EXT}}\rangle$ represent the average observed and predicted values in the external set, respectively.

Finally, the predictivity of developed models were further assessed by combining the most stringent criteria collectively suggested by Golbraikh et al. [36], Ojha et al. [37], Roy et al. [38], and Chirico and Gramatica [39]

(16) $$r^{2},q_{\mathrm{CV}}^{2},\;q^{2},q_{\mathrm{Fn}}^{2}\geq 0.70$$

(17) $$\left|r^{2}-q_{\mathrm{CV}}^{2}\right|<0.10$$

(18) $$\left(r^{2}-r_{o}^{2}\right)/r^{2}<0.10\;\mathrm{and}\;0.85\leq k\leq 1.15$$

(19) $$\left|r_{o}^{2}-{r^{\prime }}_{o}^{2}\right|<0.30$$

(20) $$r_{m}^{2}\geq 0.65$$

(21) $$\langle r_{m}^{2}\rangle \geq 0.65\;\mathrm{and}\;\Delta r_{m}^{2}<0.20$$

(22) CCC≥0.85

where *r* in Equations (18)–(21) denotes *r* and *q* in the training set and external set, respectively. $q_{\mathrm{Fn}}^{}$ in Equation (16) symbolizes $q_{F1}^{2}$, $q_{F2}^{2}$, and $q_{F3}^{2}$.

## 5. Conclusions

Intestinal permeability plays a pivotal role in systemic drug absorption that, in turn, can be of critical importance to drug efficacy. As such, intestinal permeability is one of the important drug metabolism and pharmacokinetics parameters that should be assessed in the process of drug discovery and development. An in silico model to predict the intestinal permeability can be of great value to drug discovery and development. Nevertheless, intestinal permeability is an extremely complex process that can take place through various routes, namely passive diffusion and carrier-mediated active transport. thus distinct descriptor combinations as well as various relationships are needed to depict these variations in different mechanisms. The novel machine learning-based HSVR scheme, which concurrently possesses the advantageous characteristics of a local model and a global model, namely larger coverage of applicability domain and higher degree of predictivity, respectively, was adopted in this investigation to construct a QSAR model to predict the intestinal permeability. The constructed HSVR showed great prediction accuracy for the molecules in the training set and test set, respectively, with superior predictivity and statistical significance. When subjected to a mock test by a group of molecules to mimic real challenges, the built HSVR model also performed equivalently well. In addition, the selected descriptors can render those interactions associated with passive diffusion and active transport. Accordingly, it can be asserted that this HSVR model can be utilized as an accurate and dependable predictive tool, even in the high throughput environment, to facilitate drug discovery and development by predicting the intestinal permeability of hit and lead compounds even when they are virtual. Additionally, the development of HSVR also paves the way to create more in silico models to predict oral absorption, drug stability in stomach, and bioavailability in the future.

## Figures and Tables

**Table 1:** Descriptors selected as the input of SVR models in the ensemble, the correlation coefficient (*r*) with log *P*_eff_, and their descriptions.

Descriptor	SVR A	SVR B	SVR C	*r*	Description
*μ*	X ^†^	X	X	−0.16	Dipole moment of molecule
Log *D*	X	X		0.23	Logarithm of the *n*-octanol–water partition coefficient at PH 6.5
Log *P*			X	0.22	Logarithm of the *n*-octanol–water partition coefficient
HBD		X	X	−0.07	Hydrogen-bond donor
*n*_N+O_	X			−0.29	Number of nitrogen and oxygen
Shadow-*ν*	X			0.23	Ratio of largest to smallest dimension
MR		X	X	−0.12	Sum of molar refractivity of substituents

^†^ Selected.

**Table 2:** Statistic evaluations, namely correlation coefficient (*r*^2^), maximal absolute residual (Δ_Max_), mean absolute error (MAE), standard deviation (*s*), RMSE, leave one out cross-validation correlation coefficient ($q_{\mathrm{CV}}^{2}$), and average correlation coefficient of *Y*-scrambling (<$r_{s}^{2}$>) evaluated by SVR A, SVR B, SVR C, and HSVR in the training set.

	SVR A	SVR B	SVR C	HSVR
*r* ^2^	0.62	0.83	0.79	0.93
Δ_Max_	1.18	0.91	1.42	0.50
MAE	0.33	0.25	0.15	0.16
*s*	0.26	0.15	0.27	0.08
RMSE	0.42	0.29	0.31	0.17
$q_{\mathrm{CV}}^{2}$	0.12	0.02	0.07	0.84
$\langle r_{s}^{2}\rangle$	0.02	0.02	0.02	0.02

**Table 3:** Statistic evaluations, correlation coefficients *q*^2^, $q_{F1}^{2}$, $q_{F2}^{2}$, and $q_{F3}^{2}$, concordance correlation coefficient (*CCC*), maximal absolute residual (Δ_Max_), mean absolute error (MAE), standard deviation (*s*), and RMSE evaluated by SVR A, SVR B, SVR C, and HSVR in the test set.

	SVR A	SVR B	SVR C	HSVR
*q* ^2^	0.40	0.67	0.73	0.81
$q_{F1}^{2}$	0.15	0.55	0.66	0.75
$q_{F2}^{2}$	0.15	0.55	0.66	0.75
$q_{F3}^{2}$	0.50	0.73	0.79	0.85
*CCC*	0.55	0.82	0.86	0.89
Δ_Max_	0.99	0.68	0.64	0.47
MAE	0.39	0.26	0.24	0.22
*s*	0.29	0.24	0.20	0.14
RMSE	0.47	0.35	0.30	0.26

**Table 4:** Statistic evaluations, correlation coefficients *q*^2^, $q_{F1}^{2}$, $q_{F2}^{2}$, and $q_{F3}^{2}$, concordance correlation coefficient (*CCC*), maximal absolute residual (Δ_Max_), mean absolute error (MAE), standard deviation (*s*), and RMSE evaluated by SVR A, SVR B, SVR C, and HSVR in the outlier set.

	SVR A	SVR B	SVR C	HSVR
*q* ^2^	0.34	0.63	0.72	0.83
$q_{F1}^{2}$	−0.10	0.04	0.47	0.78
$q_{F2}^{2}$	−0.11	0.04	0.47	0.78
$q_{F3}^{2}$	0.58	0.64	0.80	0.92
*CCC*	0.34	0.40	0.65	0.89
Δ_Max_	0.97	0.64	0.52	0.31
MAE	0.33	0.36	0.26	0.17
*s*	0.29	0.19	0.16	0.09
RMSE	0.43	0.40	0.30	0.19

**Table 5:** Validation verification of HSVR based on prediction performance of the molecules in the training set, test set, and outlier set.

	Training Set	Test Set	Outlier Set
$r_{o}^{2}$	0.94	0.75	0.83
*k*	1.04	0.99	0.87
${r^{\prime }}_{o}^{2}$	0.93	0.80	0.78
$r_{m}^{2}$	0.88	0.66	0.76
${r^{\prime }}_{m}^{2}$	0.90	0.74	0.67
$\langle r_{m}^{2}\rangle$	0.89	0.70	0.71
$\Delta r_{m}^{2}$	0.02	0.08	0.08
Equation (16)	X ^†^	X	X
Equation (17)	X	N/A	N/A
Equation (18)	X	X	X
Equation (19)	X	X	X
Equation (20)	X	X	X
Equation (21)	X	X	X
Equation (22)	N/A ^a^	X	X

^†^ Fulfilled; ^a^ Not available.

## Supplementary Materials

The following are available online at https://www.mdpi.com/1422-0067/21/10/3582/s1, Table S1. Selected compounds for this study; their names, SMILES strings, CAS numbers, and observed log *P*_e_ values; their predicted values by SVR A, SVR B, HSVR, and PLS; data partitions; and references; Table S2. Optimal runtime parameters for the SVR models; Figure S1. Histograms of: (A) log *P*_eff_; (B) molecular weight (MW); (C) log *D*; (D) log *P*; (E) hydrogen-bond acceptor (HBA); (F) hydrogen-bond donor (HBD); and (G) polar surface area (PSA) in the training set and test set.

## Abbreviations

QSAR	Quantitative structure–activity relationship
HSVR	Hierarchical support vector regression
ADME/Tox	Absorption, distribution, metabolism and excretion, and toxicity
BDDCS	Biopharmaceutics drug disposition classification system
SLC	Solute carrier
ABC	ATP-binding cassette
Caco-2	Colorectal adenocarcinoma
MDCK	Madin–Darby canine kidney
PAMPA	Parallel artificial membrane permeability assay
SPIP	Single-pass intestinal perfusion
