# Supplementary material for: In Silico Prediction of Intestinal Permeability by Hierarchical Support Vector Regression
Source: Int J Mol Sci. 2020 May 19;21(10):3582. doi: 10.3390/ijms21103582 (PMC7279352; doi:10.3390/ijms21103582)
Supplement: Supplementary file 1 [file ijms-21-03582-s001.zip › Supplementary/Figure S1.docx]

*In Silico* Prediction of Intestinal Absorption by Hierarchical Support Vector Regression

# Supporting information

Ming-Han Lee^1^, Giang Ta Huong^1^, Ching-Feng Weng^2^ and Max K. Leong^1,*^

^1^ Department of Chemistry, National Dong Hwa University, Shoufeng, Hualien 974301, Taiwan

^2^ Department of Basic Medical Science, Center for Transitional Medicine, Xiamen Medical College, Xiamen 361023, Fujian, China

***** Correspondence: leong@gms.ndhu.edu.tw; Tel.: +886-3-890-3609


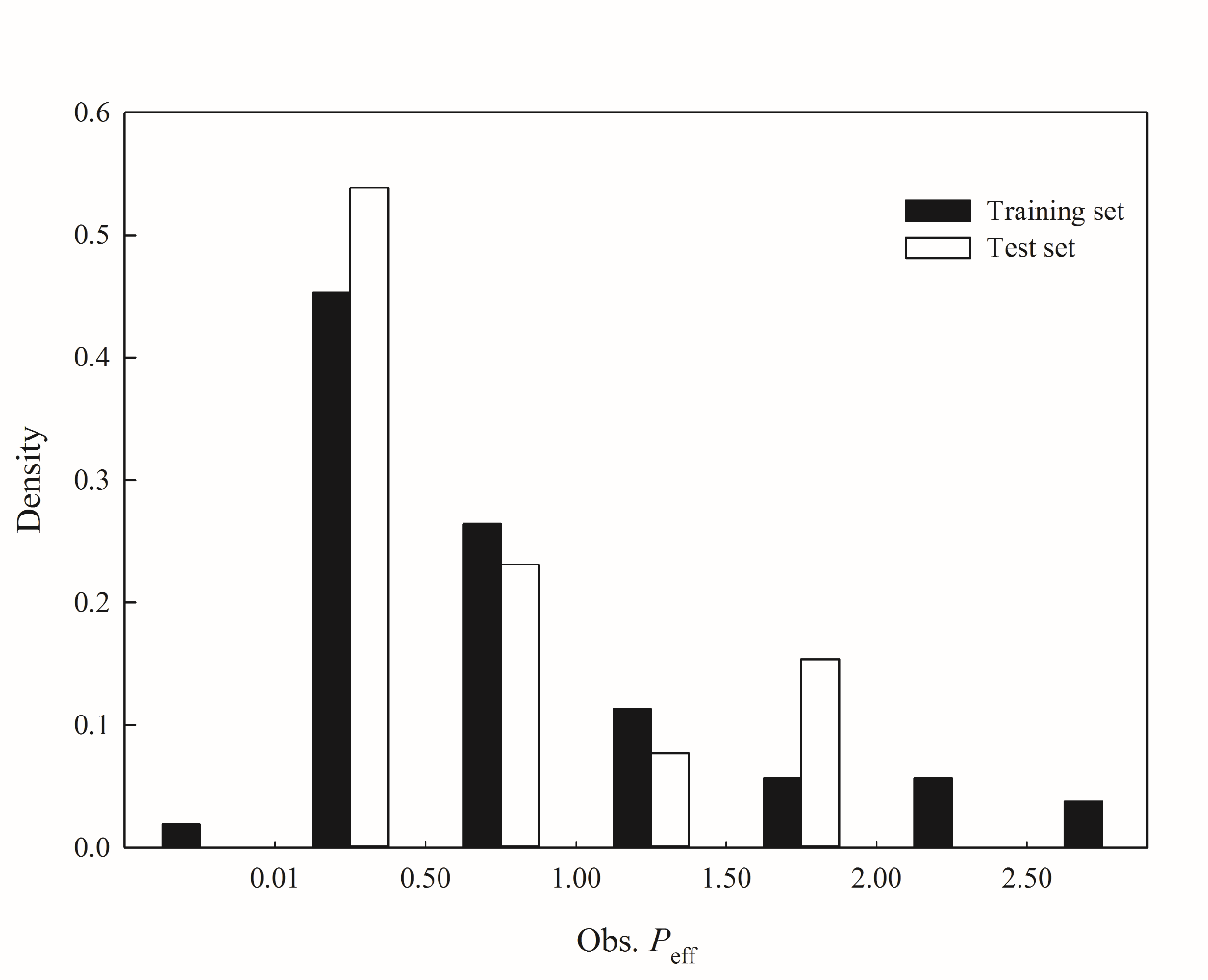


(A)


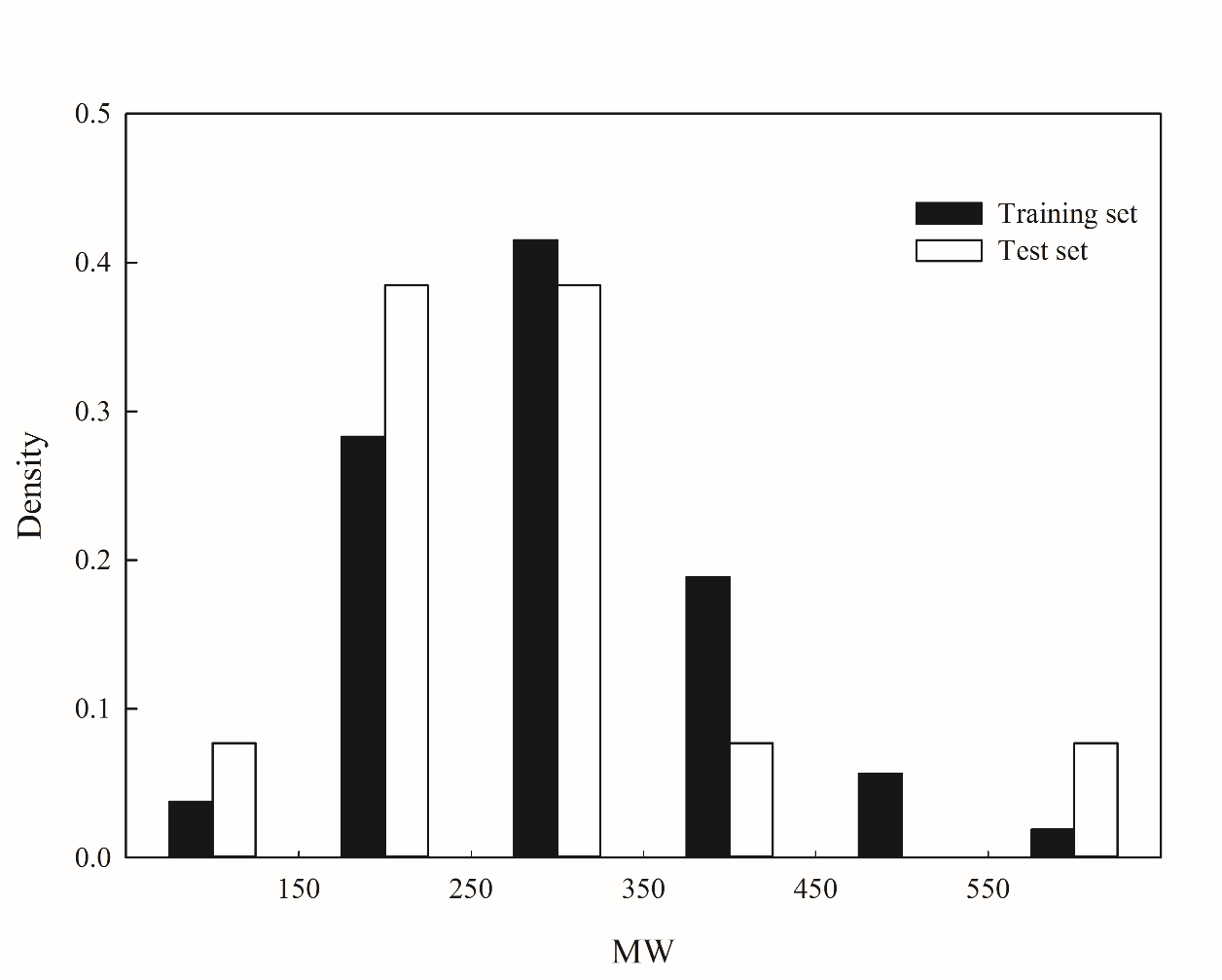


(B)


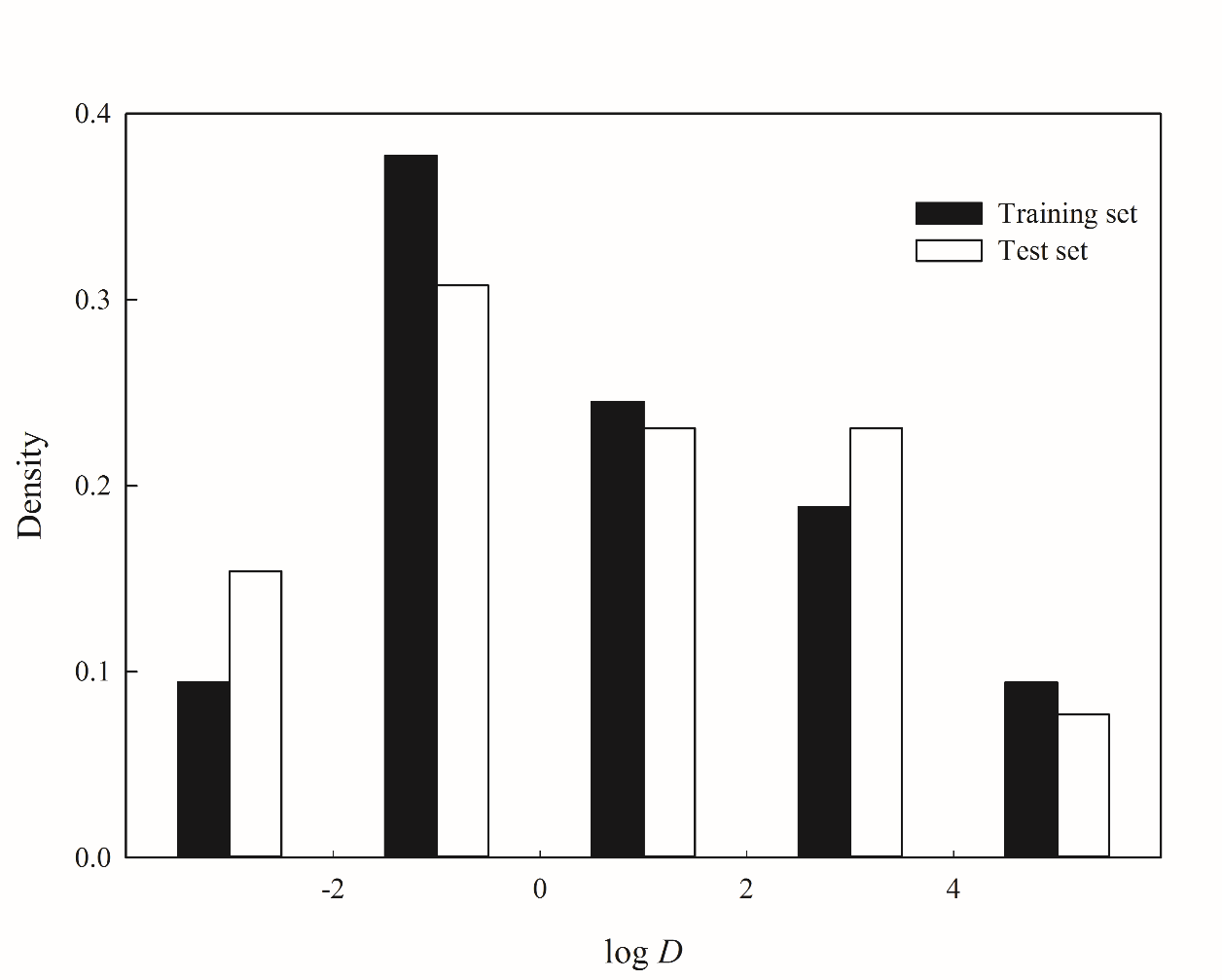


(C)


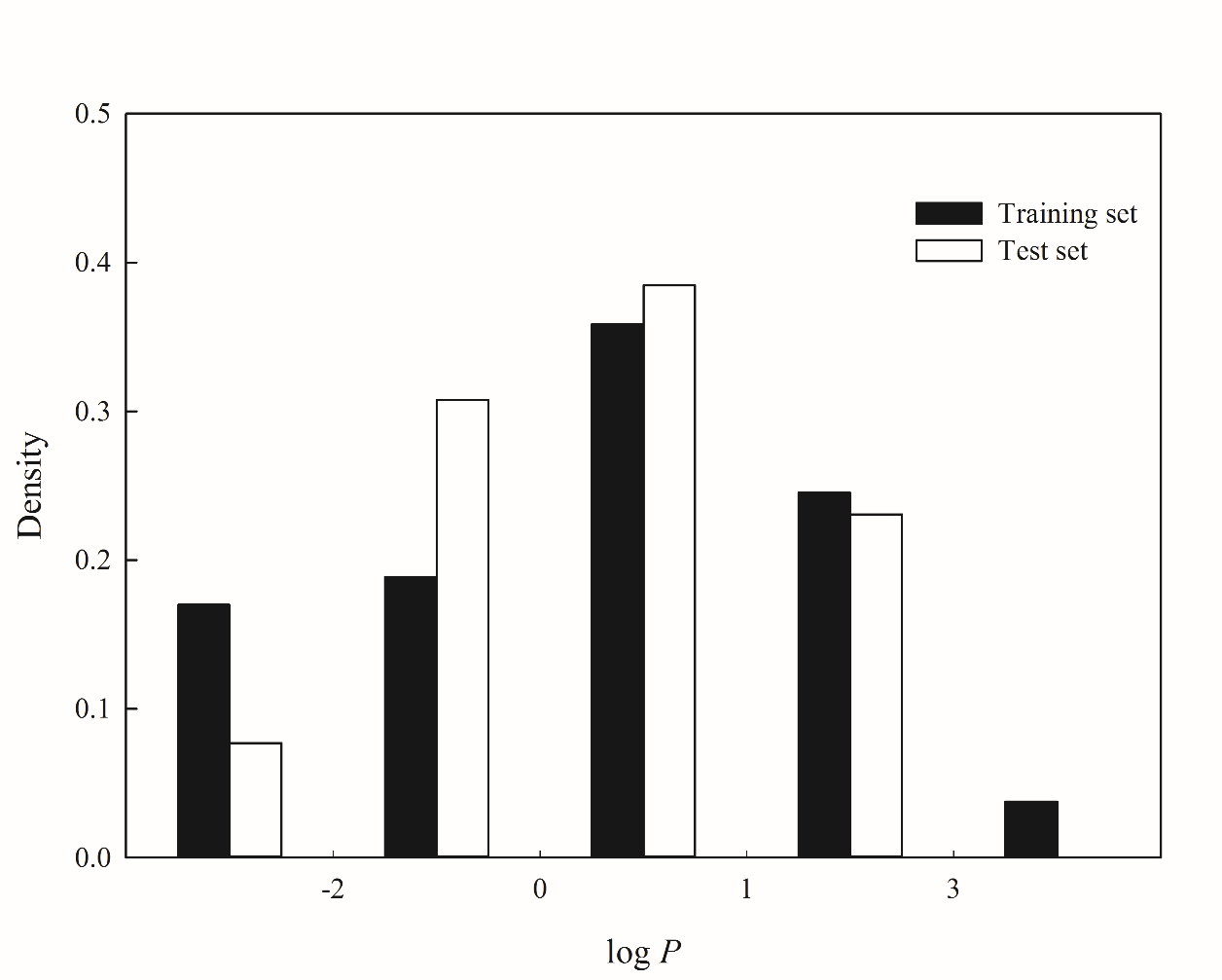


(D)


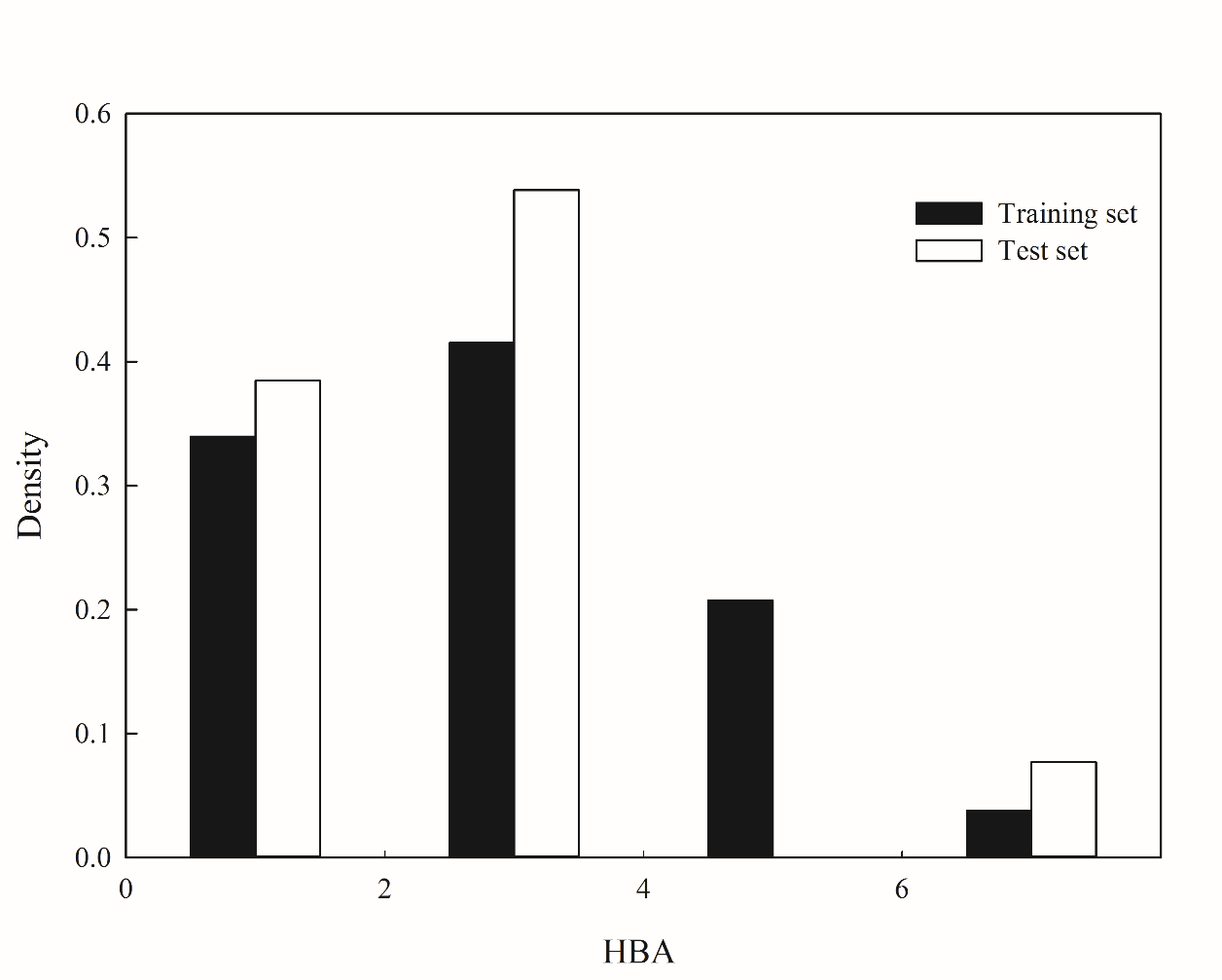


(E)


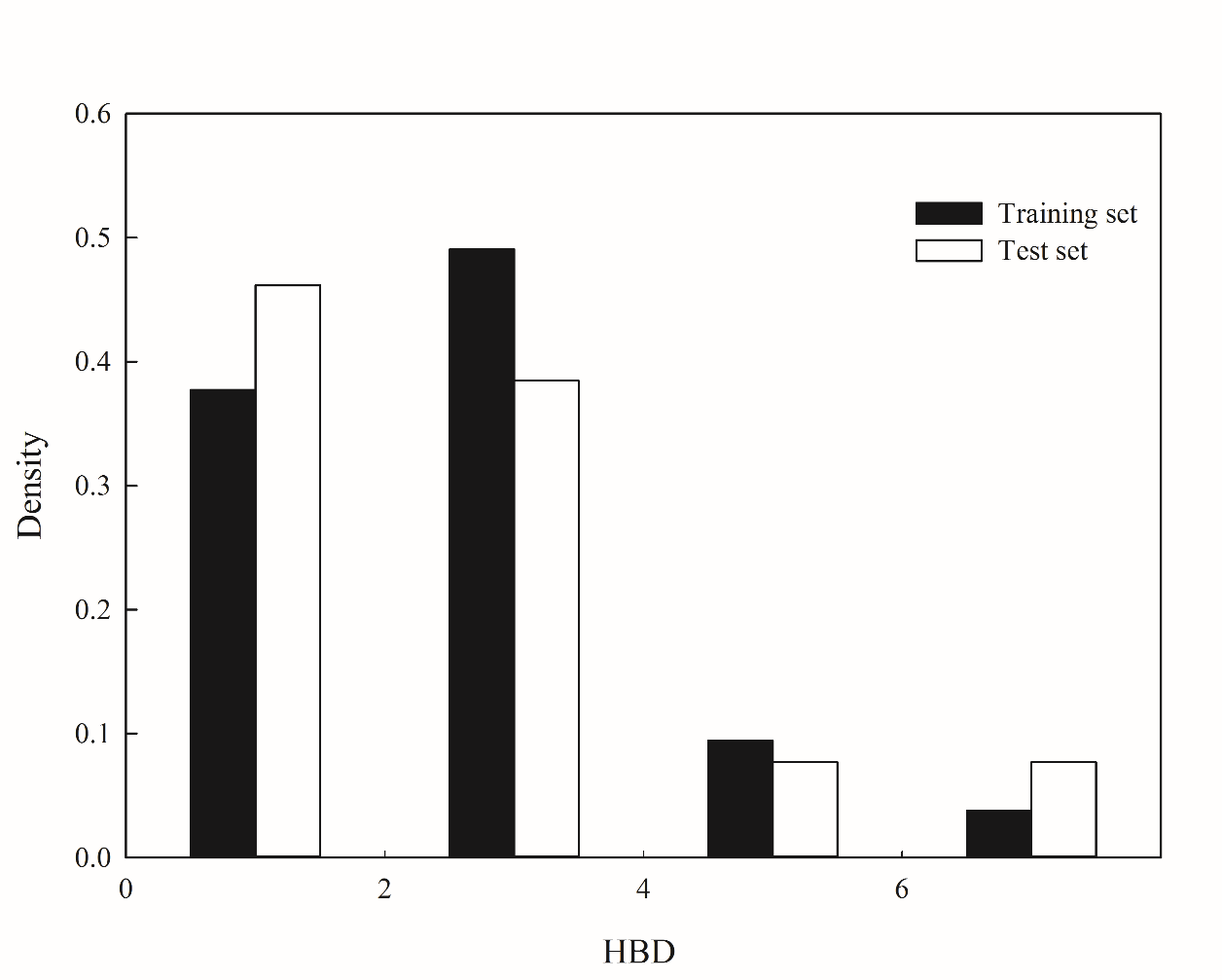


(F)


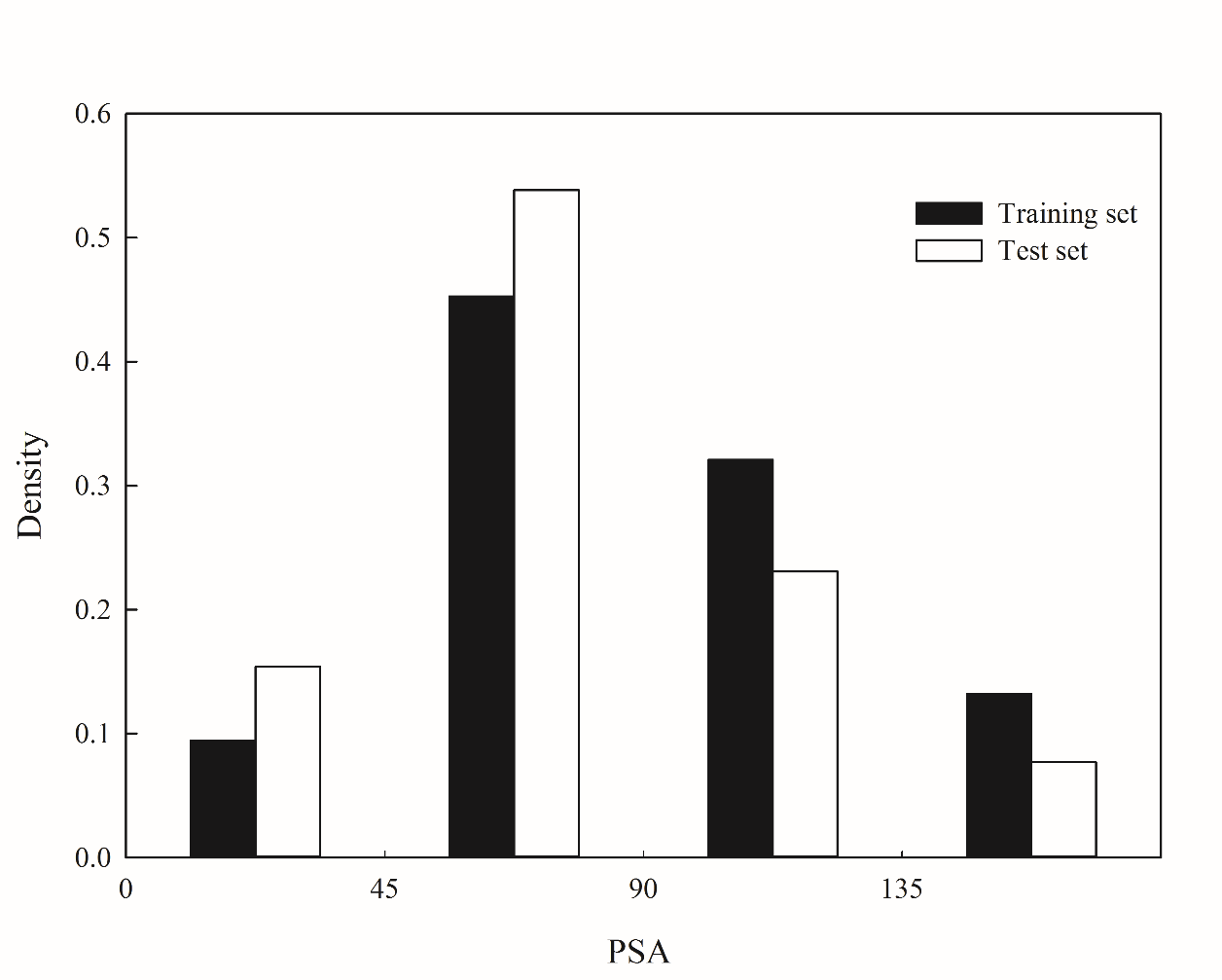


(G)

Figure S1. Histogram representation of the distributions of various descriptors for all molecules in the training set and test set. (A) log *P*_eff_, (B) molecular weight (MW), (C) log *D*, (D) log *P*, (E) hydrogen-bond acceptor (HBA), (F) hydrogen-bond donor (HBD), and (G) polar surface area (PSA) in the training set and test set.
